# Supplementary material for: Identifying the geometric catalytic active sites of crystalline cobalt oxyhydroxides for oxygen evolution reaction
Source: Nat Commun. 2022 Nov 4;13:6650. doi: 10.1038/s41467-022-34380-9 (PMC9636199; doi:10.1038/s41467-022-34380-9)
Supplement: Supplementary file 1 — Supplementary Information [file 41467_2022_34380_MOESM1_ESM.pdf]

Supporting Information for Identifying the Geometric Catalytic Active Sites of Crystalline Cobalt Oxyhydroxides for Oxygen Evolution Reaction

Sihong Wang,[1] Qu Jiang,[1] Shenghong Ju,[2] Chia-Shuo Hsu,[3] Hao-Ming Chen,[3,4] Di Zhang,[1] and Fang Song, [*,1]

[1]State Key Laboratory of Metal Matrix Composites, School of Materials Science and Engineering, Shanghai Jiao Tong University, Shanghai 200240, China

[2]China-UK Low Carbon College, Shanghai Jiao Tong University, Shanghai 201306, China

[3]Department of Chemistry, National Taiwan University, Taipei 10617, Taiwan

[4]National Synchrotron Radiation Research Center, Hsinchu 30076, Taiwan

Contents

1 Supplementary Methods 3

Method 1: *Calculation of the turn over frequency* 3

Method 2: *Structural Characterization* 3

Method 3: *X-ray absorption spectroscopy analysis* 5

Method 4: *Computational details* 6

2 Supplementary Notes 8

Note 1: *Uniformizing particles' size and their dispersion* 8

Note 2: *Purifying uniform-sized particles via differential centrifugation* 8

Note 3: *Statistical analysis of the geometric data of the particles on the flat electrode* 8

Note 4*: Calculation of surface area* 8

Note 5*: Deduction of power-law exponent relationship* 9

Supplementary Note 6*: Preparation of K^+ ion-exchange Nafion suspension* 10

3 Supplementary Figures 11

4 Supplementary Tables 40

1 Supplementary Methods

Supplementary Method 1: *Calculation of the turnover frequency*

The turnover frequency based on the electrochemical impedance spectrum (EIS) can be described as[1]:

$$TOF_{EIS} = \frac{1}{4\tau} = \frac{1}{4 \times R_{CT} \times C_{\mu}} \quad 1)$$

where τ stands for the relaxation time (s) for a charge transfer process at the catalyst–solution interface, R_{CT} stands for the pseudocapacitive charging/discharging through the faradaic resistors. C_{μ} stands for the chemical capacitance of the catalyst surface (charging/discharging the active sites).

The apparent TOF value was calculated from the equation:

$$TOF = \frac{J \times A}{4 \times F \times m} \quad 2)$$

where J is the current density at a given overpotential (for example, $\eta = 300$ mV), A is the surface area of the electrode, F is the Faraday constant, and m is the number of moles of cobalt atoms on the electrode. The value of m was determined based on the mass loading or the facet area.

Supplementary Method 2: *Structural Characterization*

XRD

The crystal structure of catalysts was determined using X-ray Diffraction. A Brucker D8 DaVinci diffractometer equipped with D/tex Ultra silicon trip detector and Cu Kα radiation (λ = 1.5418 Å) was used. Sample powders were dispersed in ethanol and drop-casting on the zero-background holder made of single-crystal silicon to fill a 4 mm diameter × 0.1 mm deep groove. The angle was varied between 10° to 90° with a step size of 0.05° at a scan rate of 5 degrees per min.

XPS

XPS measurements of the dried CoOOH NR and NS samples were carried out with an AXIS Ultra DLD photoelectron spectrometer (Kratos, UK). XPS spectra were recorded using a monochromated Al-Kα X-ray source (hν = 1486.6 eV) operated at 15 kV and 14 mA and a spot size of 300 μm. The detector was at normal incidence to the sample surface enabling analysis up to a depth of 50–100 Å. Survey spectra were collected using a pass energy of 160 eV and a step size of 0.7 eV, to check the sample purity. High-resolution spectra were collected at 10 eV pass energy using a step size of 0.025 eV.

Electron Microscopy

The structural characterization was carried out by using Scanning Electron Microscopy (SEM) and Transmission Electron Microscopy (TEM). TEM experiments were performed in a Talos F200X (FEI, USA) equipped with a cold field-emission electron gun using an accelerating voltage of 200 kV. Powder samples were prepared in ethanol and sonicated for 2 min before drop-casting on the 400-mesh copper grid. The morphologies of NR and NS particles are also investigated using MAGNA SEM (TESCAN). The high-throughput SEM (Navigator-100 FBT) was carried out to characterize the particles dispersed on the plate electrode.

AFM

Atomic force microscopy (AFM) measurements were carried out using a Bio FastScan scanning probe microscope (Bruker, Dimension Icon & FastScan Bio, Germany). For ex-situ AFM, CoOOH NS was dispersed on the silicon substrates by spin-coating, and washing with deionized water and ethanol several times. A homemade Teflon cell was used for in-situ AFM. A piece of 1×1 cm HOPG was chosen as the substrate because of its atomically flat surface. The HOPG that was modified with CoOOH NS (see electrode fabrication below) was mounted on the bottom of the cell and was sealed with 704 silica gel. The HOPG was attached to the Cu wire by Cu tap, which is also covered with 703 silica gel to keep it isolated from the solution. Pt wire and standard Ag/AgCl electrodes were used as the counter electrode and reference electrode, respectively. The electrochemical AFM measurement was conducted in 0.1 M KOH. A CHI 760E model Electrochemical Workstation (Shanghai) was used to accomplish the electrochemical measurement during the AFM imaging procedure. The AFM images were collected on a Bruker Dimension Icon using ScanAsyst-fluid probe (Bruker) in Liquid-ScanAsyst mode.

Raman

The Raman spectra were recorded on a confocal Raman microscope with a laser wavelength of 532 nm excitation wavelength. The in situ Raman spectra were collected in a three-electrode cell. An In-via microspore with a water-immersion objective (Olympus LUMFL, 60×, numerical aperture = 1.10) was used to focus and collect the incident and scattered laser light during electrochemical measurements. A 0.013 mm thin optically transparent Teflon film was used to protect the objective from the corrosive KOH electrolyte. To keep the light path stable and eliminate the interference of refraction, a drop of water was added between the Teflon film and the objective lens. The Raman frequencies were calibrated using a Si wafer before the measurement. The catalyst/GC electrode was attached to the customized cell, whose counter and reference electrodes are Pt foil and Hg/HgO, respectively. The cell was injected with 0.1 M KOH (~50 mL) and connected to a CHI 760E model Electrochemical Workstation (Shanghai). Raman spectra were acquired at low power (around 0.2 mW) to prevent the formation of Co_3O_4. Each Raman spectrum was recorded with a resolution of 1 cm^{-1} by averaging five scans, each of 2 s duration. A typical CV scan was performed at a rate of 0.5 $mV{\cdot}s^{-1}$ during the acquisition of Raman

spectra. The obtained spectra were background-subtracted to make the Raman peaks clearer.

BET Surface Area

Nitrogen sorption isotherms were measured on an Autosorb iQ2 gas sorption analyzer (Quantachrome, UK) at 77 K. Before the measurement, powder samples were degassed at 110 °C for 20 h. The BET surface areas (A_{BET}) of the CoOOH NR and NS were calculated from the N_2 adsorption/desorption data fitted to the Brunauer–Emmett–Teller (BET) model.

Supplementary Method 3: *X-ray absorption spectroscopy analysis*

X-ray absorption spectroscopy (XAS) measurements including X-ray absorption near-edge spectra (XANES) and extended X-ray absorption fine structure (EXAFS) were carried out for the K-edge of CoOOH samples. XAS data were collected in total-fluorescence-yield mode at beamline 01C1 at the National Synchrotron Radiation Research Center in Taiwan. The electron storage ring was operated at 3.0 GeV with a constant current of ~400 mA. The incident beam energy was monochromatized using a Si (111) double-crystal monochromator. The scan range was kept in an energy range of 7600-8300 eV for Co K-edge. The data collected were normalized to the incoming incident photon flux and processed with the Athena software from the IFEFFIT package. An E_0 value of 7709.0 eV was used to calibrate all data with respect to the first inflection point of the absorption K-edge of cobalt foil. The spectra were obtained after subtracting the baseline of pre-edge and normalizing that of post-edge. EXAFS analysis was conducted using Fourier transform on k^3-weighted EXAFS oscillations to evaluate the contribution of each bond pair to the Fourier transform peak. To obtain quantitative structural parameters around central atoms, least-squares curve parameter fitting was performed using the ARTEMIS module of IFEFFIT software packages.

EXAFS fittings

EXAFS curve fitting was performed with the ARTEMIS module of IFEFFIT software packages using ab initio-calculated phases and amplitudes. These ab initio phases and amplitudes were used in the EXAFS equation:

$$\chi(k) = S_0^2 \sum_j \frac{N_j}{kR_j^2} f_{eff_j}(\pi, k, R_j) e^{-2\sigma_j^2 k^2} e^{-2R_j/\lambda_j(k)} \sin(2kR_j + \Phi_{ij}(k)) \quad 3)$$

The distance from the neighboring atoms to the central atoms is divided into *j* shells, with all atoms with the same atomic number and distance from the central atom grouped into a single shell. Within each shell, the coordination number N_j denotes the number of neighboring atoms in shell *j* at a distance of R_j from the central atom. $f_{eff_j}(\pi, k, R_j)$ is the ab initio amplitude function for shell *j*, and the Debye-Waller term $e^{2\sigma_j^2 k^2}$ accounts for the damping result from a static and thermal disorder in absorber-backscatterer distances. The

mean free path term $e^{-2R_j/\lambda_j(k)}$ reflects loss due to inelastic scattering, where $\lambda_j(k)$ is the electron mean free path. The oscillations in the EXAFS spectrum are reflected in the sinusoidal term $\sin(2kR_j + \Phi_j(k))$, where $\Phi_{ij}(k)$ is the ab initio phase function for shell j. S_0^2 is an amplitude reduction factor due to shake-up/shake-off processes at the central atom(s). The EXAFS equation was used to fit the experimental data using CN, R, and the EXAFS Debye-Waller factor (DW; σ^2) as variable parameters. For the energy (eV) to wave vector (k, $Å^{-1}$) axis conversion, the S_0^2used in the fitting was based on the CoOOH NS and fixed to be 0.81 for CoOOH NR. All fittings were performed in the R space.

Supplementary Method 4: *Computational details*

The following mechanism of OER proposed by Nørskov and co-workers was employed in this work.

$$4OH^- + * \rightarrow *OH + 3OH^- + e^- \quad 4)$$

$$*OH + 3OH^- \rightarrow *O + H_2O + 2OH^- + e^- \quad 5)$$

$$*O + H_2O + 2OH^- \rightarrow *OOH + H^+ + e^- \quad 6)$$

$$*OOH + OH^- \rightarrow * + O_2 + H_2O + e^- \quad 7)$$

* represents the active sites at the surface of CoOOH. *OH, *O, and *OOH represent the intermediate species adsorbed on the active sites. The energy of (H^+ + e^-) was replaced with $1/2E(H_2)$.

For each step, the standard-state free energy changes (ΔG) at 298.15 K are calculated as

$$\Delta G_1 = G_{OH} - eU + \Delta G_{H^+}(pH) \quad 8)$$

$$\Delta G_2 = \Delta G_O - \Delta G_{OH} - eU + \Delta G_{H^+}(pH) \quad 9)$$

$$\Delta G_3 = \Delta G_{OOH} - \Delta G_O - eU + \Delta G_{H^+}(pH) \quad 10)$$

$$\Delta G_4 = 4.92[eV] - \Delta G_{OOH} - eU + \Delta G_{H^+}(pH) \quad 11)$$

where U is the potential measured against normal hydrogen electrode (NHE) at standard conditions (T = 298.15 K, P = 1 bar, pH = 0). The free energy change of the protons relative to the above-specified electrode at non-zero pH is represented by the Nernst equation as $\Delta G_{H^+}(pH) = -k_B T \ln(10) \times pH$.

To avoid the calculation of the O_2 bond energy, which is difficult to determine accurately within GGA-DFT, the calculation of ΔG_4 is completed by fixing the sum of $\Delta G_{1\sim4}$ to the negative of experimental Gibbs free energy of the formation of two water molecules $-2\Delta G_{H_2O}^{exp} = 4 \times 1.23 = 4.92 eV$.

The Gibbs free energies of the intermediates *OH, *O, and *OOH were calculated according to

$$\Delta G_i = \Delta E_i + \Delta ZPE_i + T\Delta S_i \cdot \quad 12)$$

where ΔZPE is the zero-point energy that can be obtained by frequency analysis. The adsorption energy (Δ*E*) of three radicals (*O, *OH, *OOH) can be calculated by the following equations:

$$\Delta E_{OH} = E(*OH) - E(*) - \left[E(H_2O) - \frac{1}{2}E(H_2)\right] \quad 13)$$

$$\Delta E_O = \Delta E(O*) - E(*) - [E(H_2O) - E(H_2)] \quad 14)$$

$$\Delta E_{OOH} = \Delta E(*OOH) - E(*) - \left[2E(H_2O) - \frac{3}{2}E(H_2)\right] \quad 15)$$

Based on the above DFT calculation, the theoretical overpotential is then readily defined as:

$$\eta = \max[\Delta G_1, \Delta G_2, \Delta G_3, \Delta G_4]/e - 1.23 \ [V] \quad 16)$$

2 Supplementary Notes

Supplementary Note 1: *Uniformizing particles' size and their dispersion*

The sizes of the $Co(OH)_2$ nanosheets were uniformized by a hydrothermal treatment[2]. Typically, about 10 mg of the as-prepared $Co(OH)_2$ nanosheets were dispersed in 40 mL of deionized water. 0.1 mL of hydrazine hydrate ($N_2H_4 \cdot H_2O$, $80_{wt}\%$, Sigma-Aldrich) was added to prevent the $Co(OH)_2$ from being oxidized during hydrothermal treatment. The aqueous suspension was transferred into a Teflon-lined autoclave for a hydrothermal reaction at 120 °C for 4 hours. In addition to uniformizing particles' size, this hydrothermal treatment impedes particles' aggregates, leading to monodispersed particles on substrates.

Supplementary Note 2: *Purifying uniform-sized particles via differential centrifugation*

To remove any possible fragments or aggregates, differential centrifugation was performed. Typically, the $Co(OH)_2$ particles were dispersed in formamide. For L-$Co(OH)_2$, the suspension was centrifuged at 20 × g for 5 min to collect the precipitates at the bottom. For ML- $Co(OH)_2$, the suspension was centrifuged at 100 × g for 5 min, and then the resultant supernatant was centrifuged at 240 × g for 5 min. The precipitates at the bottom were collected. For M- $Co(OH)_2$, the suspension was centrifuged at 500 × g for 5 min, and then the supernatant was centrifuged at 1800 × g for 5 min. The precipitates at the bottom were collected as the products. For S- $Co(OH)_2$, the suspension was centrifuged at 4800 × g for 2 min and the precipitates at the bottom were collected.

Supplementary Note 3: *Statistical analysis of the geometric data of the particles on the flat electrode*

The high through-put SEM was used to acquire digital images of the sample deposited on the surface of flat electrodes. Image-Pro Plus (IPP) software, which can analyze digital images according to their different color models, was applied to determine the geometric parameters of particles for the subsequent statistical analysis. For each sample, the statistical analysis involved 10 randomly chosen regions at the size of 0.2 mm × 0.2 mm.

Supplementary Note 4: *Calculation of surface area*

The calculation of the surface area is based on the structural models shown in Supplementary Fig. S29. The basal size (d) of NS and the length (l) of nanorods were determined by analyzing SEM images of nanoparticles on flat electrodes (see Note 3). The thickness/height (h) of NS was measured by AFM. An average height 'h' of ~60 nm was used to calculate the surface area of NS, as all the samples have a similar thickness. The basal surface area S_b, lateral surface area S_l and overall surface area $S_{overall}$ were calculated according to the following equations:

$$S_b = n \times 2 \times \frac{3\sqrt{3}}{2} \times (\frac{d}{2})^2 = \frac{3\sqrt{3}}{4} nd^2 \quad 17)$$

$$S_l = n \times 6 \times \frac{d}{2} \times h = 3ndh \quad 18)$$

$$S_{overall} = S_b + S_l = \frac{3\sqrt{3}}{4} nd^2 + 3ndh \quad 19)$$

$$V_{overall} = S_b h = \frac{3\sqrt{3}}{8} nd^2 h \quad 20)$$

where $V_{overall}$ is the overall volume; 'n' is the total number of nanoparticles, assuming that all the particles have the same size, for the sake of simplicity. Since CoOOH NR can be regarded as NS stacking along the lateral direction, their surface area can be calculated by replacing the thickness 'h' (in the above equations) with the length 'l' of CoOOH NR.

Supplementary Note 5: *Deduction of power-law exponent relationship*

In most cases, the catalytic activity was probed at a similar mass loading 'm'. The relation between the mass and the particle size can be given by the equation:

$$m = \rho V_{overall} = \rho S_b h = \frac{3\sqrt{3}}{8} n\rho d^2 h \quad 21)$$

where 'm' and 'ρ' are the mass and density of catalysts, respectively.

On the premise that the mass is the same, we can calculate the basal, lateral, and overall surface area at the mass loading of 'm' with the following equations:

$$S_b = \frac{m}{\rho h} \quad 22)$$

$$S_l = \frac{8m}{\sqrt{3}\rho d} \quad 23)$$

$$S_{overall} = S_b + S_l += \frac{m}{\rho h} + \frac{8m}{\sqrt{3}\rho d} \quad 24)$$

They suggest that, in the case of the same mass loading, the overall lateral plane area of all the crystals is proportional to the reciprocal of the crystal diameter (1/d), whereas the basal plane surface area is only relevant to the average height of the crystals.

The overall current densities of NR and NS are dependent on the intrinsic activity and the surface area of each specific plane (basal and lateral plane here), following equation 1:

$$J_{overall} = J_b + J_l = j_b \times S_b + j_l \times S_l = {j_b m}/{\rho h} + {8 j_l m}/{\sqrt{3}\rho d} \quad 25)$$

where $J_{overall}$, J_b, and J_l are the overall, basal, and lateral current density; j_b and j_l are the specific current densities on basal and lateral planes.

We then discussed the overall catalytic activity of CoOOH NS as below:

i) In case only the basal plane is active (that is $j_b \neq 0$ but $j_l = 0$), the overall current density is calculated by the equation:

$$J_{overall} = {j_b m}/{\rho h} \quad 26)$$

It suggests that the activity is a constant value (the thickness of NS is the same from sample to sample).

ii) In case only the lateral plane is active (that is j_b=0 but $j_l \neq 0$), the overall current density is calculated by the equation:

$$J_{overall} = {8j_l m}/{\sqrt{3}\rho d} \quad 27)$$

It suggests a linear dependence of activity on the reciprocal basal size (1/d) (crossing the origin point).

iii) In case both are active (that is $j_b \neq 0$ but $j_l \neq 0$), the overall current density is calculated by the equation:

$$J_{overall} = {j_b m}/{\rho h} + {8j_l m}/{\sqrt{3}\rho d} \quad 28)$$

It suggests a linear dependence of activity on the reciprocal basal size (1/d), but the intercept value should be large (>0).

Supplementary Note 6: *Preparation of K^+ ion-exchange Nafion suspension*

A ~3.33 wt.% K^+ ion-exchanged Nafion solution was used as an immobilizing binder[3]. Typically, 1 mL of 0.1 M KOH aqueous solution was mixed with 2 mL 5 wt.% proton-type Nafion suspension. The resulting Nafion suspension shows a pH ~11 after mixing, which would ensure the stability of our CoOOH catalysts.

3 Supplementary Figures

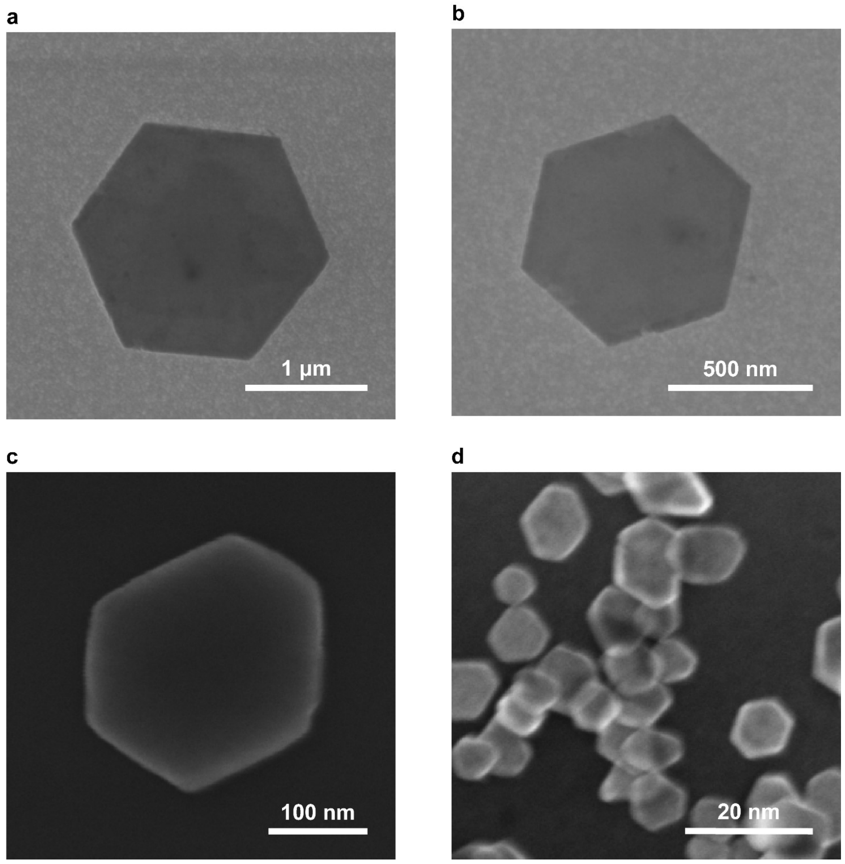

Supplementary Figure 1| SEM images of $Co(OH)_2$ nanosheets. (a) L-$Co(OH)_2$. (b) ML-$Co(OH)_2$. (c) M-$Co(OH)_2$. (d) S-$Co(OH)_2$.

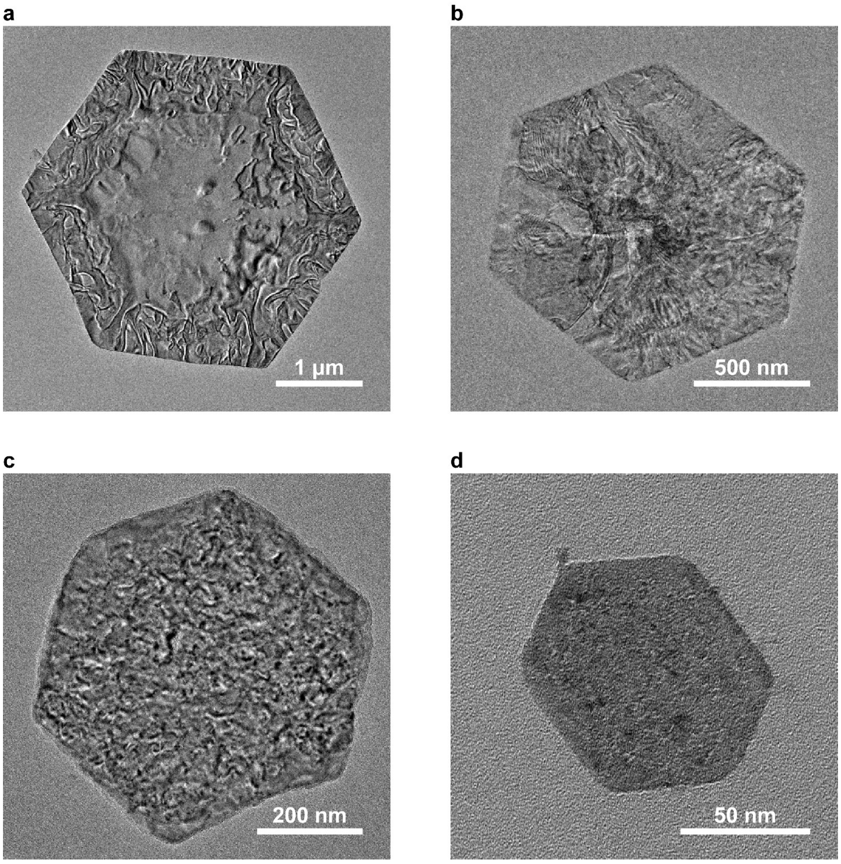

Supplementary Figure 2| TEM images of $Co(OH)_2$ nanosheets. (a) L-$Co(OH)_2$. (b) ML-$Co(OH)_2$. (c) M-$Co(OH)_2$. (d) S-$Co(OH)_2$.

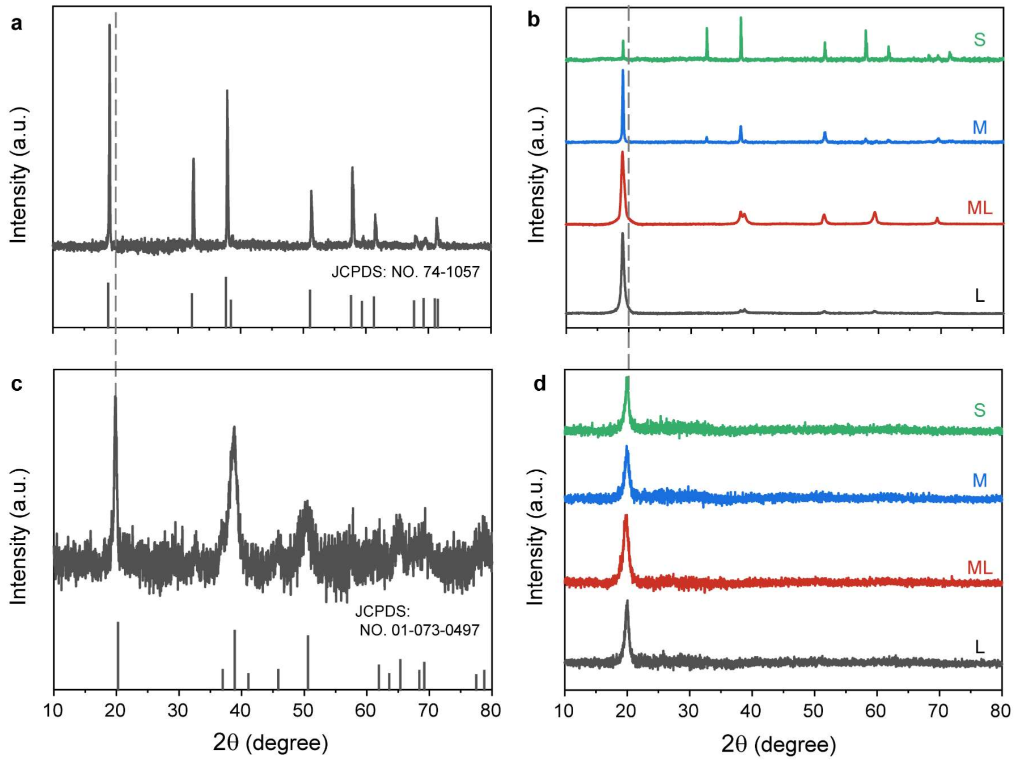

Supplementary Figure 3| XRD patterns of $Co(OH)_2$ and CoOOH samples. (a) $Co(OH)_2$ nanorods; (b) $Co(OH)_2$ nanosheets of different sizes. NS-S is displayed in green, NS-M in blue, NS-ML in red, and NS-L in black; (c) CoOOH nanorods; (d) CoOOH nanosheets with different sizes. NS-S is displayed in green, NS-M in blue, NS-ML in red, and NS-L in black. The prominent diffraction peaks at 20.3°, 38.2°, and 50.3° correspond to the (003), (012), and (015) facets reflections of the hexagonal rhomb-centered crystal structure of CoOOH (space group of $R\bar{3}m$, JPCD: 01-073-0497), respectively. The overall topochemical conversion of $Co(OH)_2$ to CoOOH was evidenced by the (003) peak shift from ca. 19° to 20.3°. The XRD intensities indicate arbitrary units (a.u.). Source data are provided as a Source Data file.

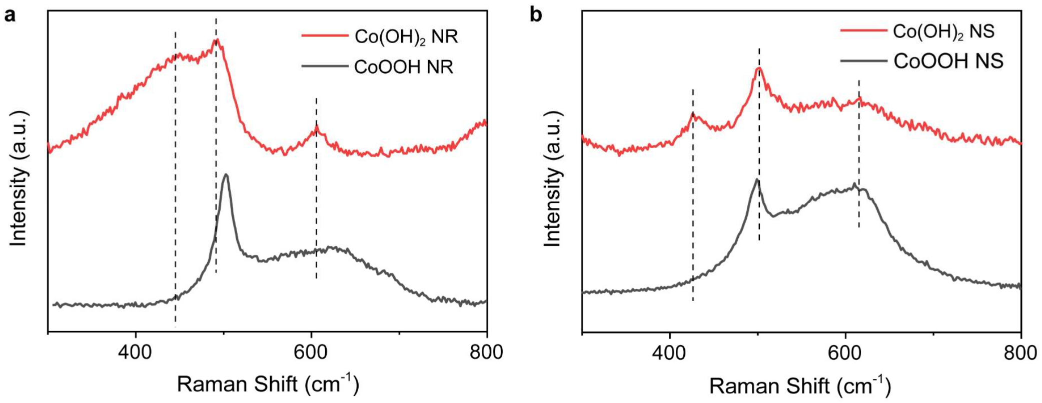

Supplementary Figure 4| Raman spectra of $Co(OH)_2$ and CoOOH samples. (a) $Co(OH)_2$ NR (red) and CoOOH NR (black); (b) $Co(OH)_2$ NS (red) and CoOOH NS. The Raman intensities indicate arbitrary units (a.u.). Source data are provided as a Source Data file.

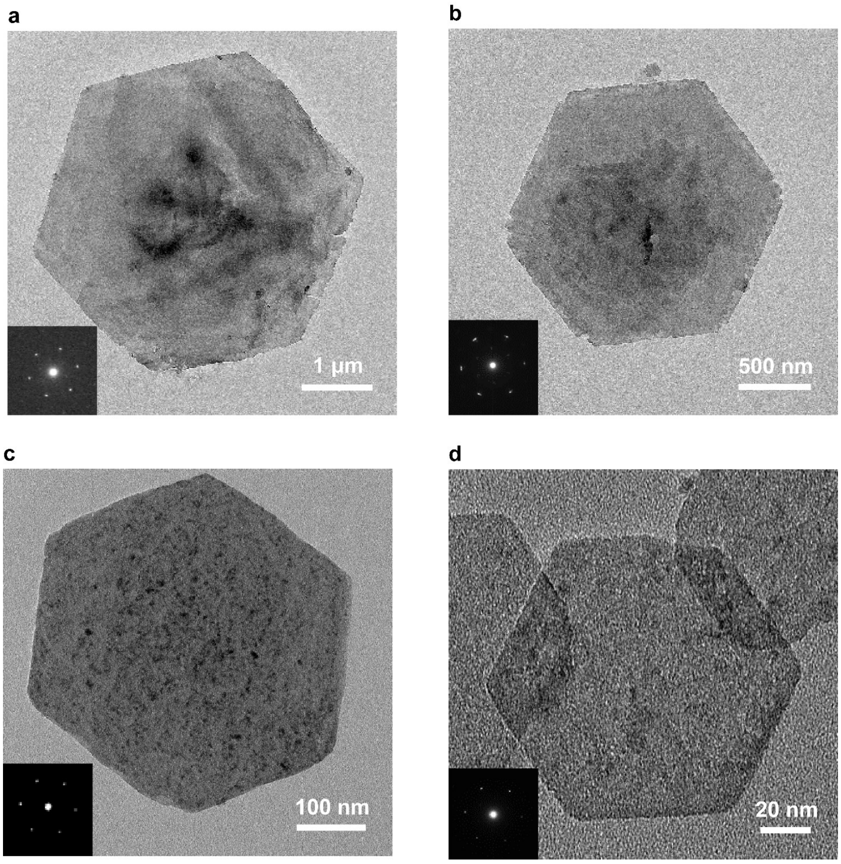

Supplementary Figure 5| TEM images of CoOOH nanosheets. (a) L-NS. (b) ML-NS. (c) M-NS. (d) S-NS. Insets are the corresponding SAED patterns.

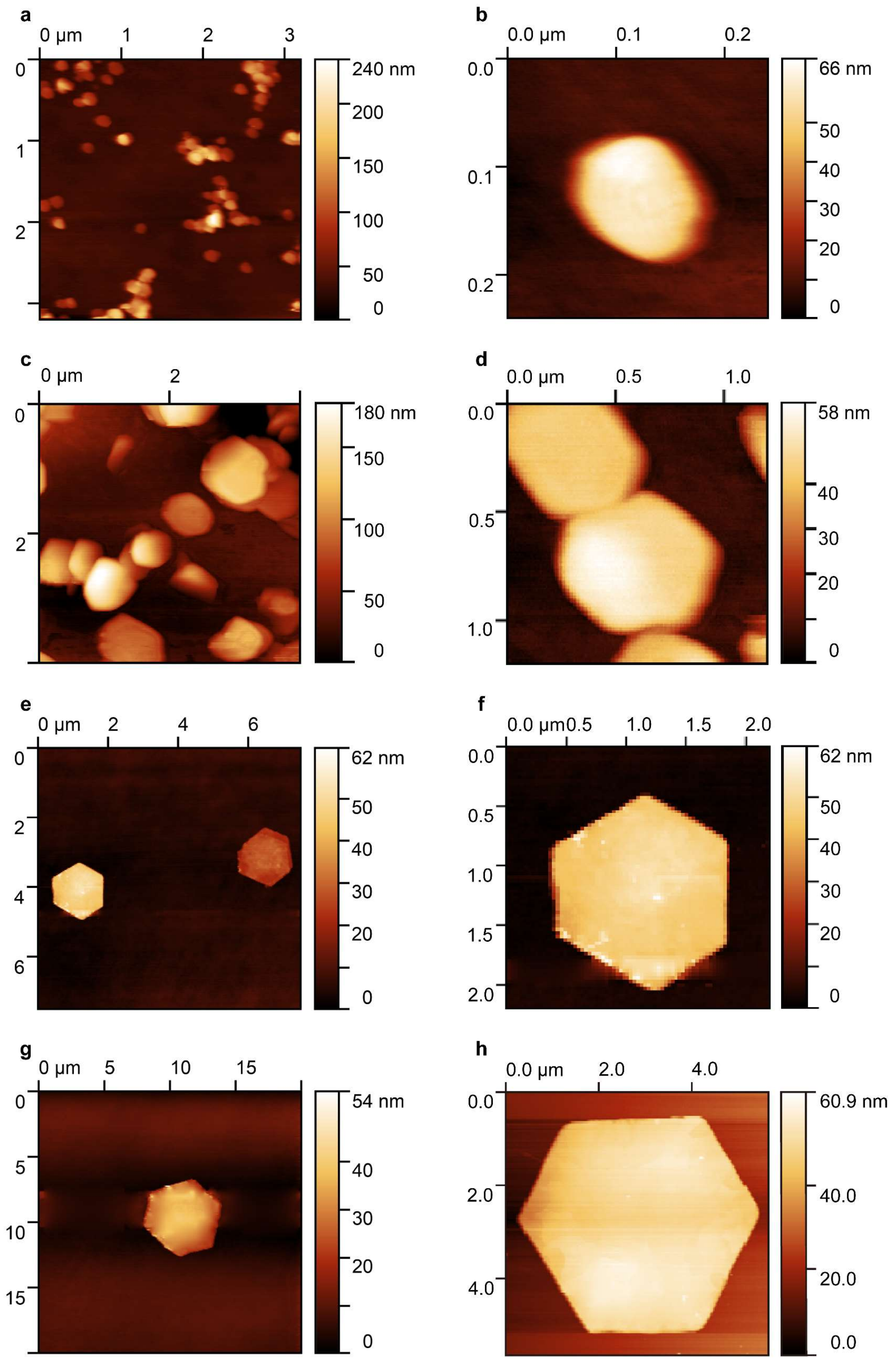

Supplementary Figure 6| AFM images of CoOOH NS. (a-b) CoOOH-S; (c-d) CoOOH-ML; (e-f) CoOOH-M; (g-h) CoOOH-L.

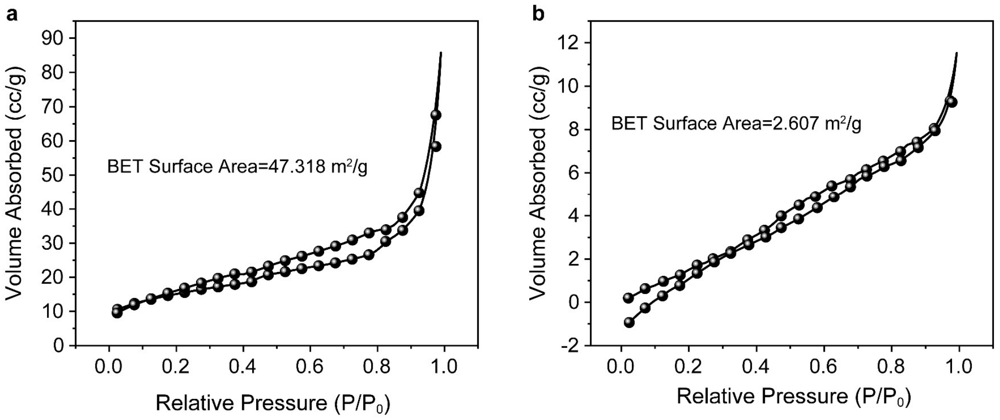

Supplementary Figure 7| Nitrogen adsorption/desorption isotherms of CoOOH samples. (a) CoOOH NS. (b) CoOOH NR. Source data are provided as a Source Data file.

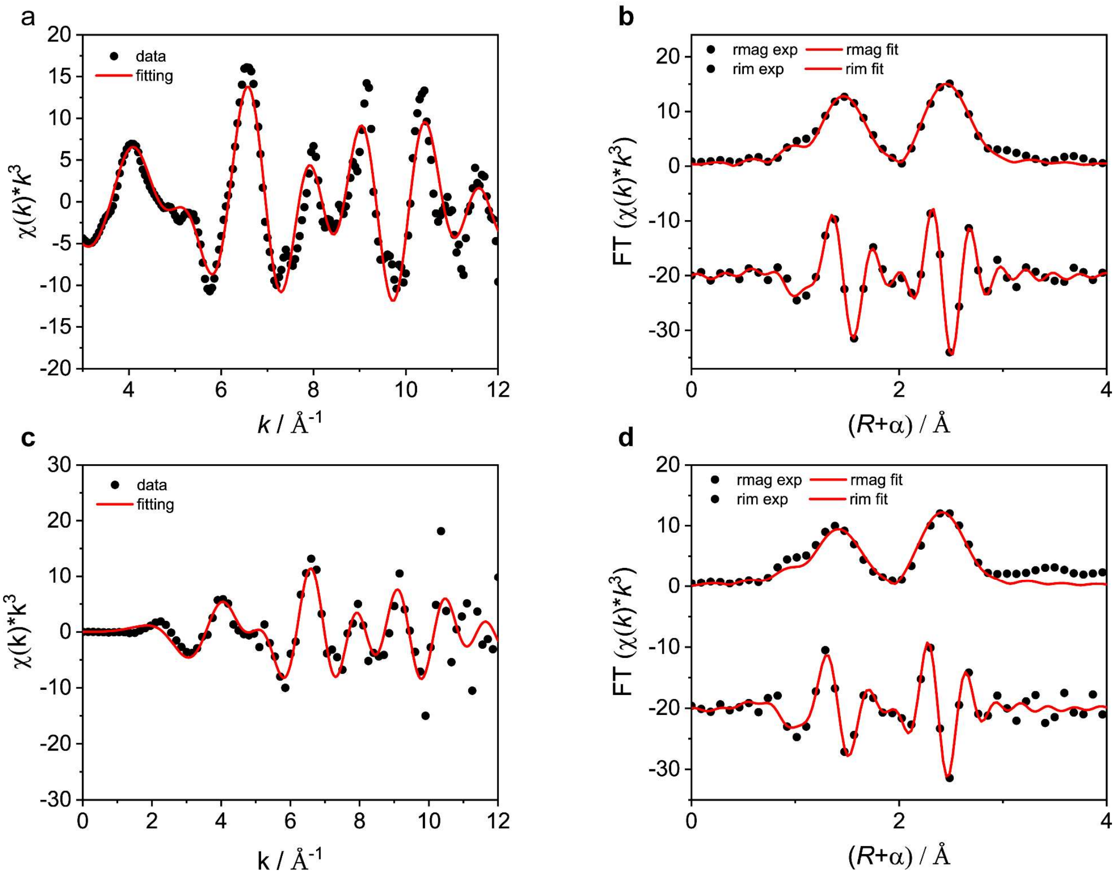

Supplementary Figure 8| (a) R space and (b) K space curve fitting on CoOOH NS; (c) R space and (d) K space curve fitting on CoOOH NR. Source data are provided as a Source Data file.

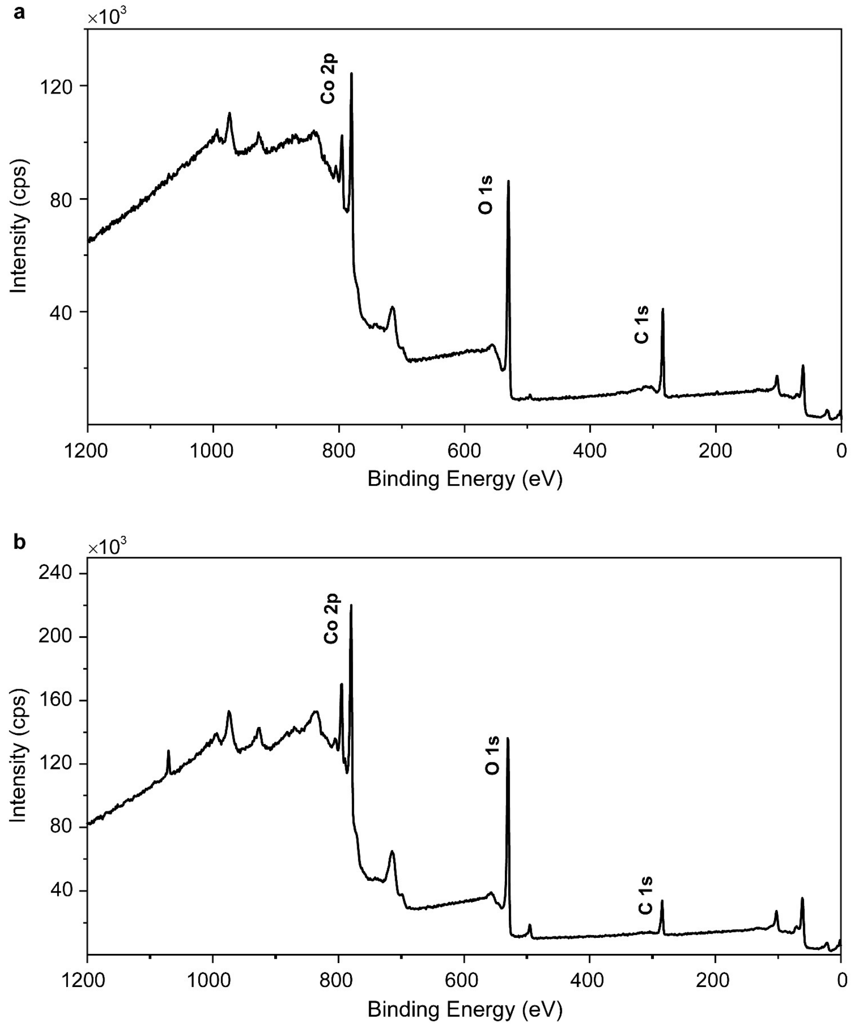

Supplementary Figure 9| XPS spectra of CoOOH nanosheets and nanorods. (a) CoOOH NS. (b) CoOOH NR. Source data are provided as a Source Data file.

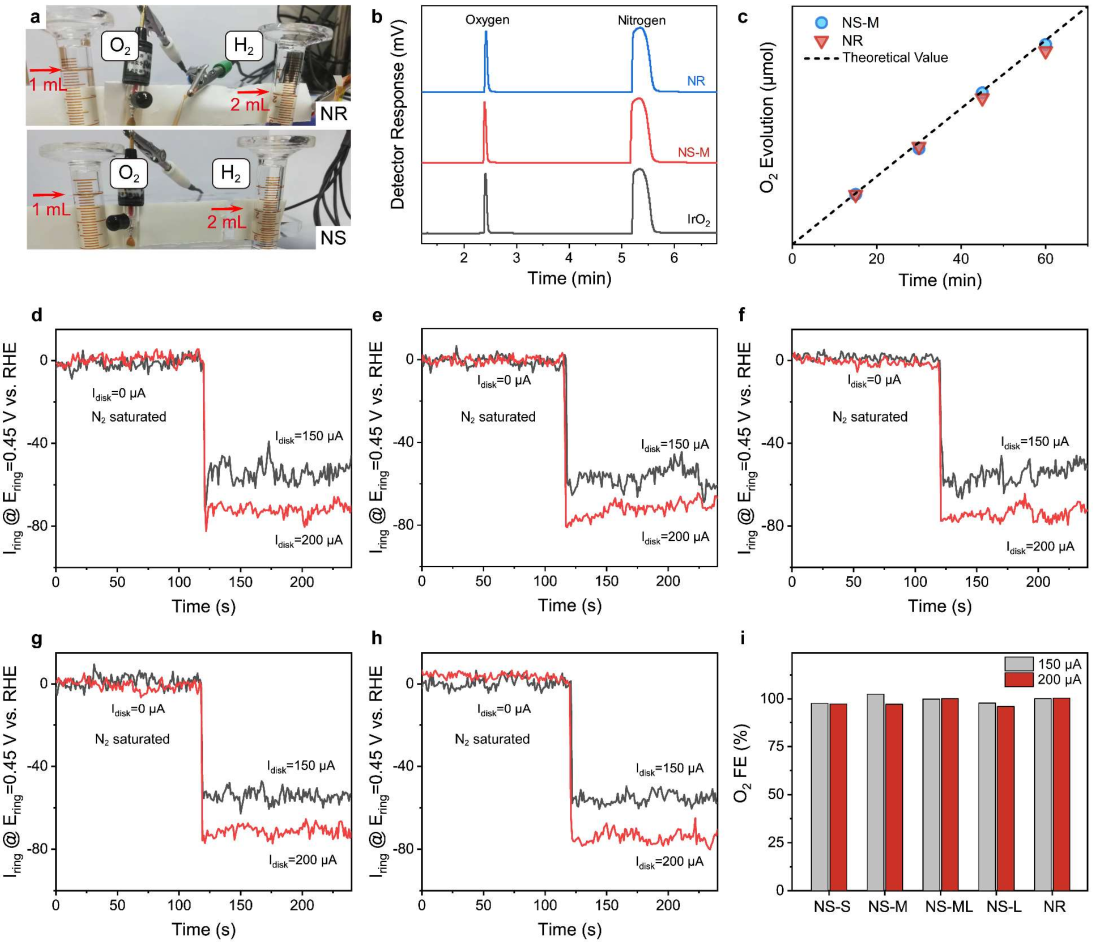

Supplementary Figure 10| Measurement of the Faradaic efficiency. (a) Photographs of water splitting electrolyzer during the electrolysis (drainage method). (b) Gas chromatography curves of gaseous products from the OER catalyzed by NR (blue), NS-M (red), and commercial IrO_2 (black) catalyst loaded on carbon fiber paper. (c) Experimental and theoretical volumes of O_2 gases during the electrolysis. (d-h) Ring current of (d) NS-S, (e) NS-M, (f) NS-ML, (g) NS-L, (h) NR on an RRDE (1500 r. p.m.) in N_2-saturated 1 M KOH solution (ring potential is set to 0.38 V and disk current is set to 150 µA (black) and 200 µA (red)). (i) Calculated faradaic efficiency of NS and NR via RRDE method (I_{disk}=150 µA (grey) and I_{disk}=200 µA (red)). Source data are provided as a Source Data file.

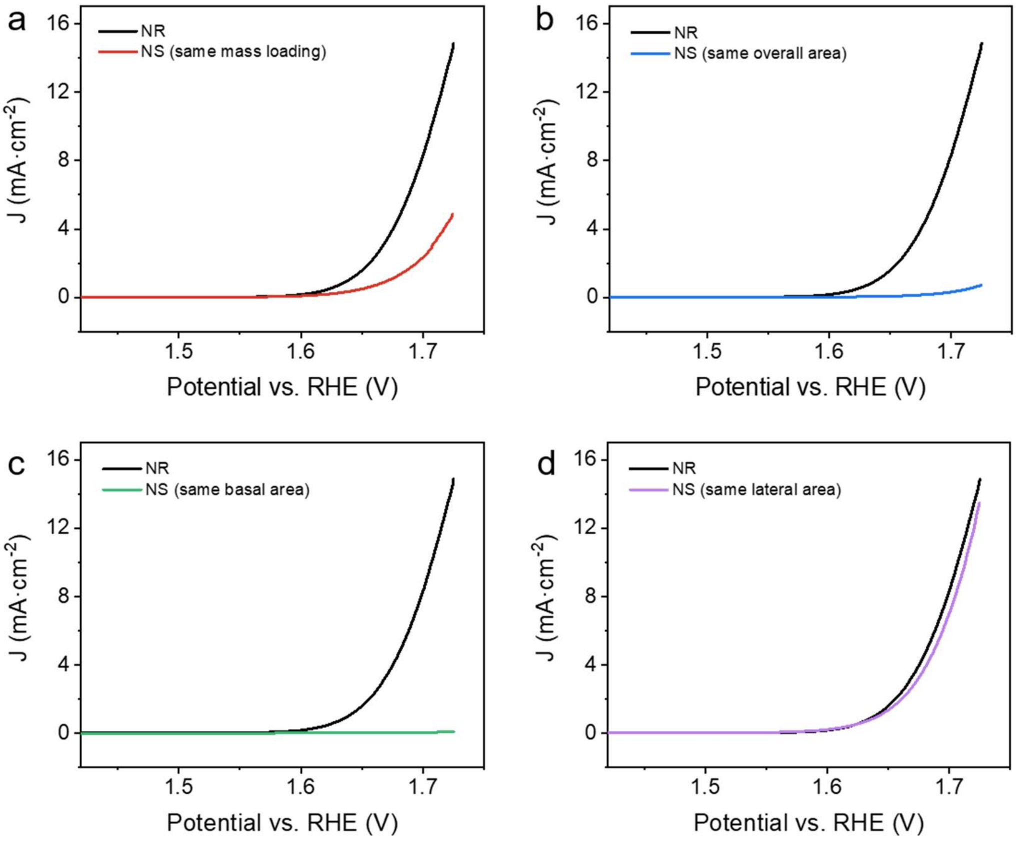

Supplementary Figure 11| LSV curves of NR and NS-ML samples at controlled mass loadings. Polarization curves of NR (black) in comparison with NS-ML in case they have (a) the same mass (NS is displayed in red), (b) overall surface area (NS is displayed in blue), (c) basal plane area (NS is displayed in green), and (d) lateral plane area (NS is displayed in purple). The area of NS-ML was tuned by manipulating the loading. Source data are provided as a Source Data file.

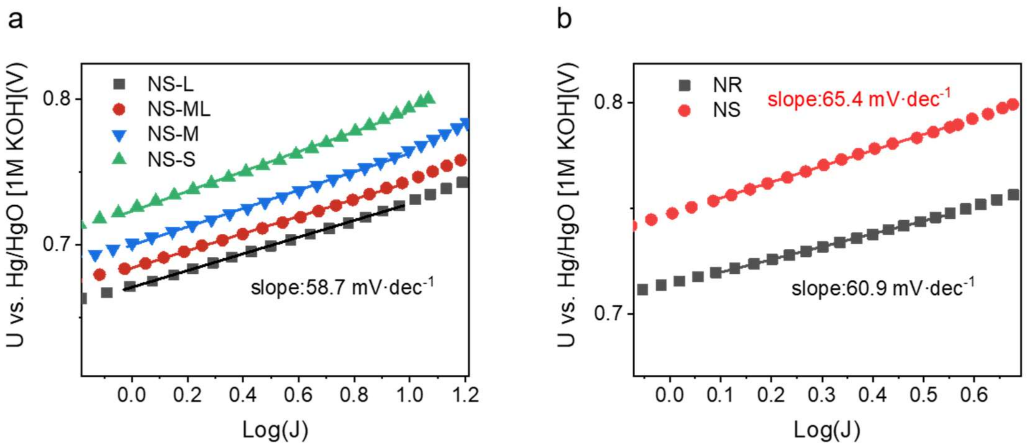

Supplementary Figure 12| Tafel plots of CoOOH NR and NS. (a) Tafel plotting of NS-L (black), NS-ML (red), NS-M (blue) and NS-S (green). (b) Tafel plotting of CoOOH NR (black) and CoOOH NS-ML (red). Source data are provided as a Source Data file.

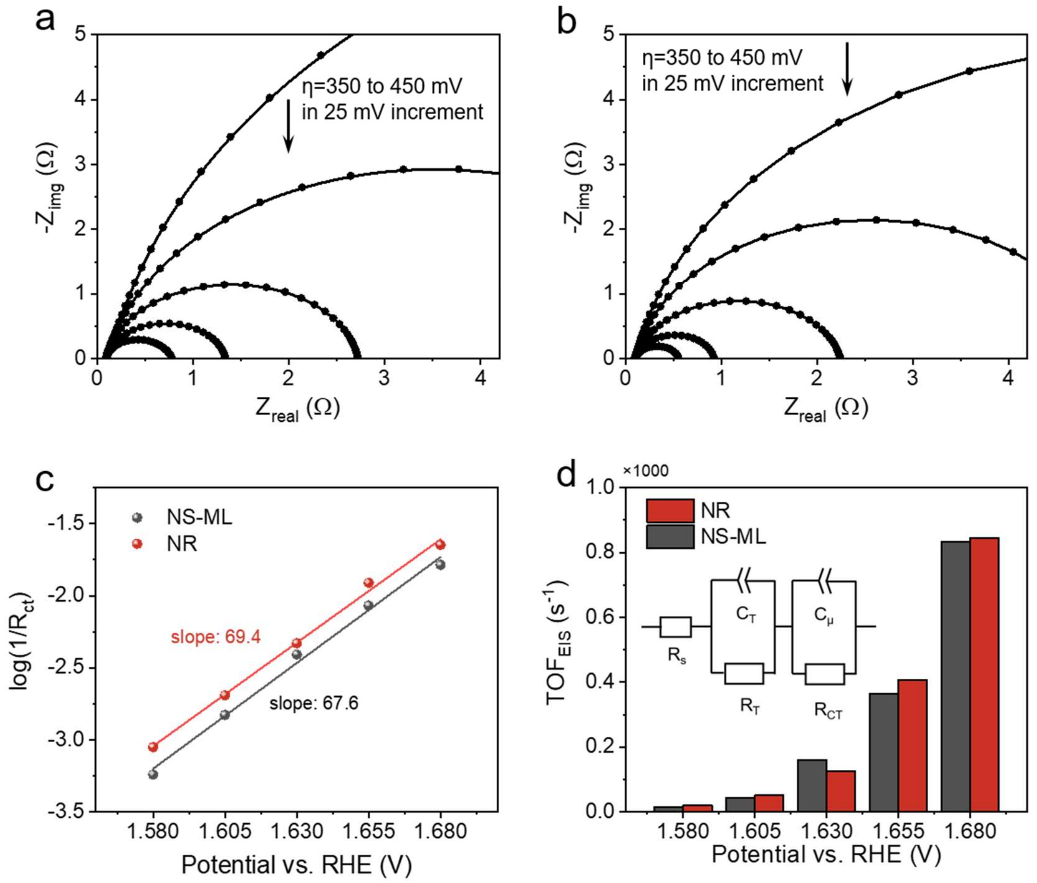

Supplementary Figure 13 | Electrochemical impedance spectroscopy analysis of CoOOH NR and CoOOH NS. (a-b) The Nyquist plots for the impedance response of (a) NS-ML and (b) NR. (c) Plot of log (R_{CT}^{-1}) vs. potentials, showing Tafel slopes of c.a. 60 mV/dec. NS-ML is displayed in black and NR in red. (d) TOF_{EIS} of NS-ML and NR at a set of applied potentials. NS-ML is displayed in grey and NR in red. The inset in (d) shows the equivalent circuit model for EIS fitting. R_s stands for series resistance; R_T stands for the catalyst's charge-transport resistance; R_{CT} stands for the charge-transfer resistance at the catalyst/solution interface; Cμ stands for the chemical capacitance. Source data are provided as a Source Data file.

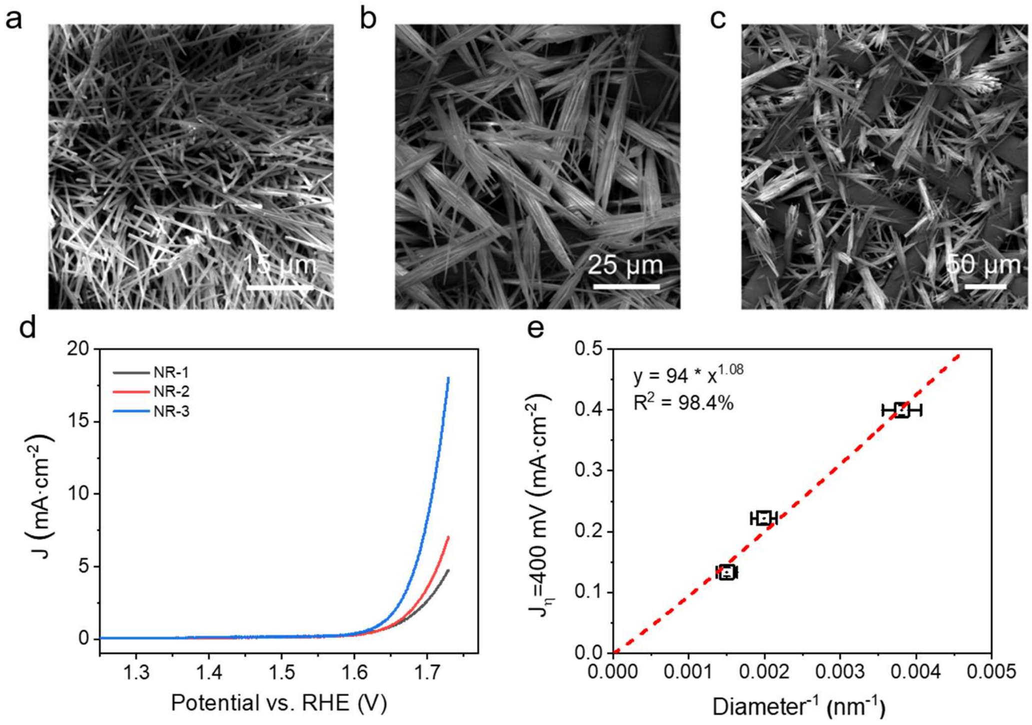

Supplementary Figure 14| Microstructures of CoOOH NR and their catalytic activities. (a-c) SEM images of (a) NR-1 with a basal facet size of ~800 nm. (b) NR-2 with a basal facet size of ~3 μm. (c) NR-3 with a basal facet size of 6.5 μm. (d) LSV curves of CoOOH NR-1 (black), NR-2 (red), and NR-3 (blue). (e) Linear fitting of the current densities at 450 mV against the reciprocal diameter 1/d of NR. The absence of deviation from linear fitting (n=1, and $J_0 = 0$) should be due to the good dispersion of NR, as well as the good electric conductivity. Standard errors for activities were calculated from the standard deviations from three measurements, and that for areas were calculated from the standard deviations of the measured size of nanorods. Source data are provided as a Source Data file.

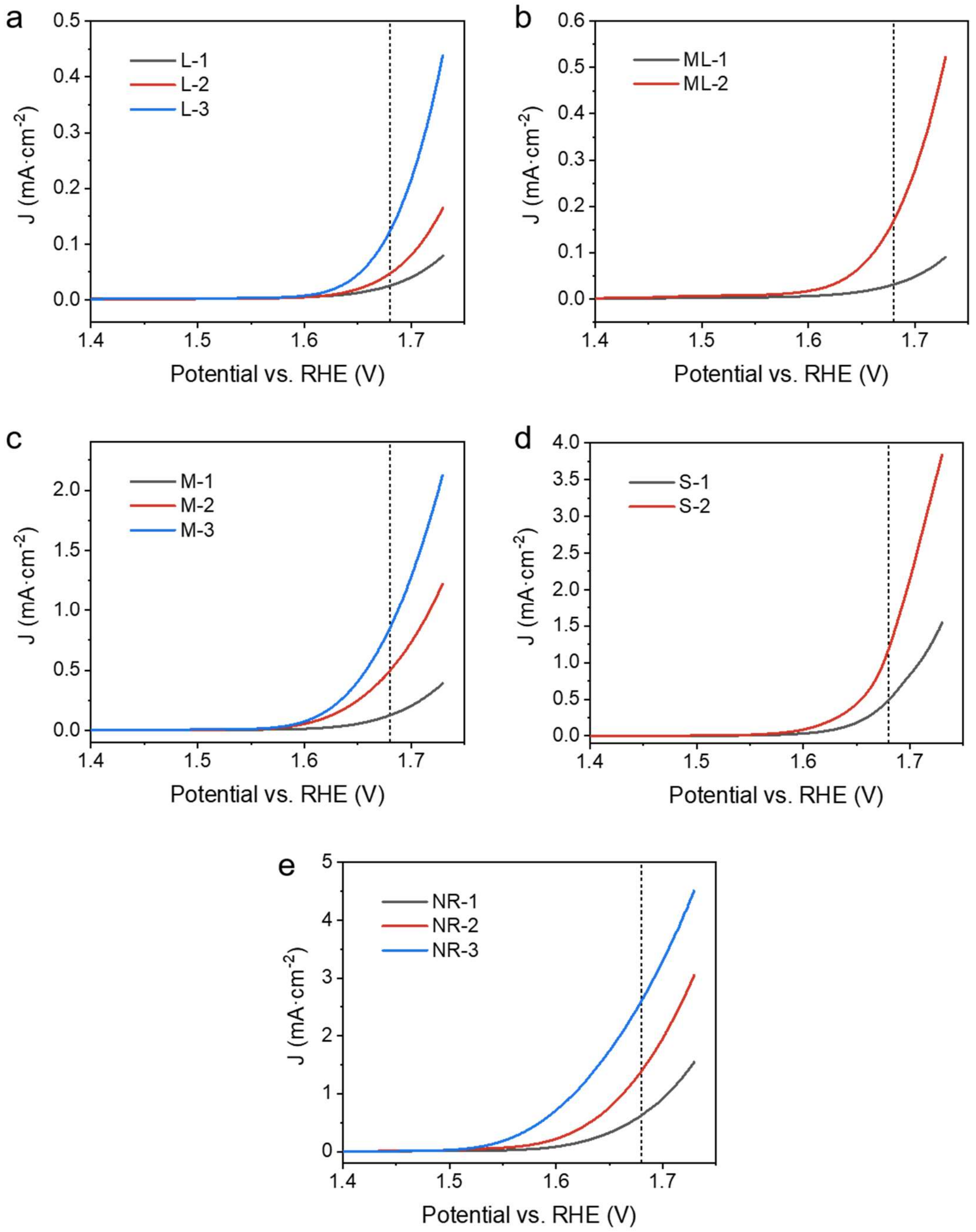

Supplementary Figure 15| LSV curves of the sub-monolayers of CoOOH samples. (a) CoOOH NS-L; (b) CoOOH NS-M; (c) CoOOH NS-ML; (d) CoOOH NS-S; (e) CoOOH NR. Source data are provided as a Source Data file.

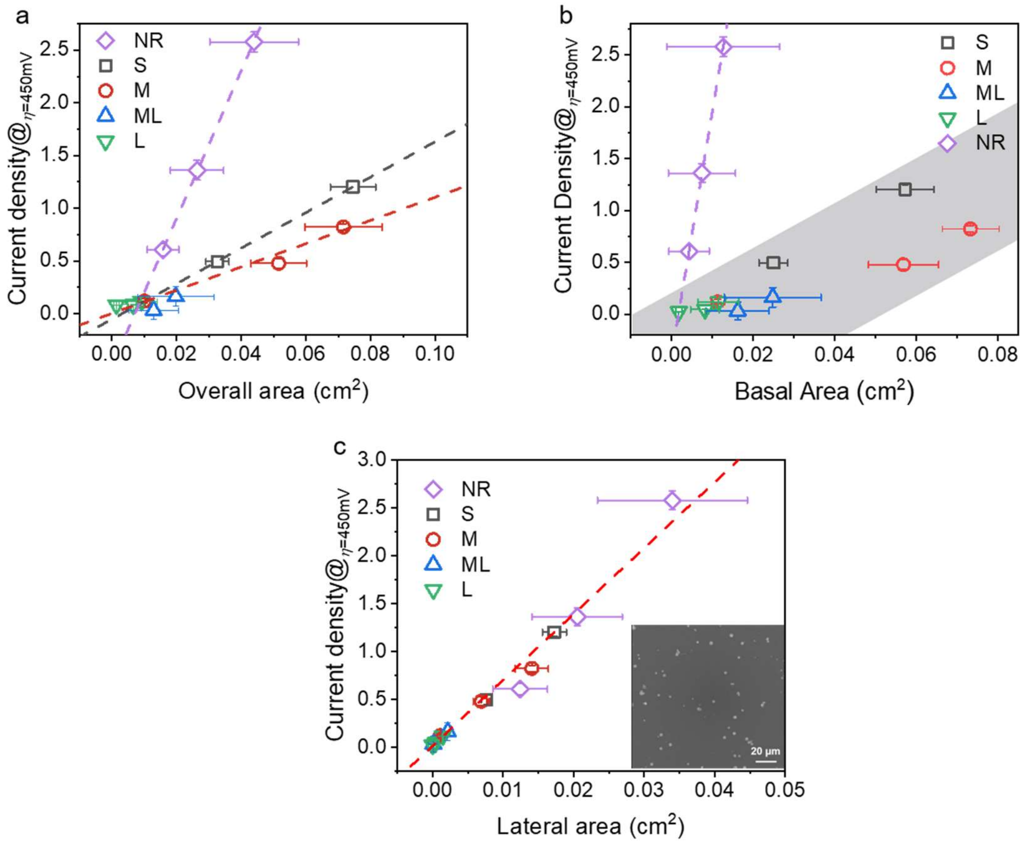

Supplementary Figure 16| The current densities of spin-coated CoOOH samples plotted against (a) overall area, (b) basal surface area, and (c) lateral surface area. It is noteworthy that the coverage of NS of large size (NS-ML and NS-L) are relatively limited due to the severe overlap of large-sized nanosheets at higher coverage. The inset in (c) is the SEM image of NS spin-coated carbon electrodes for statistical analysis of the geometric factors. Standard errors for activities were calculated from the standard deviations from three measurements, and that for areas were calculated from the standard deviations of the measured particle sizes. Source data are provided as a Source Data file.

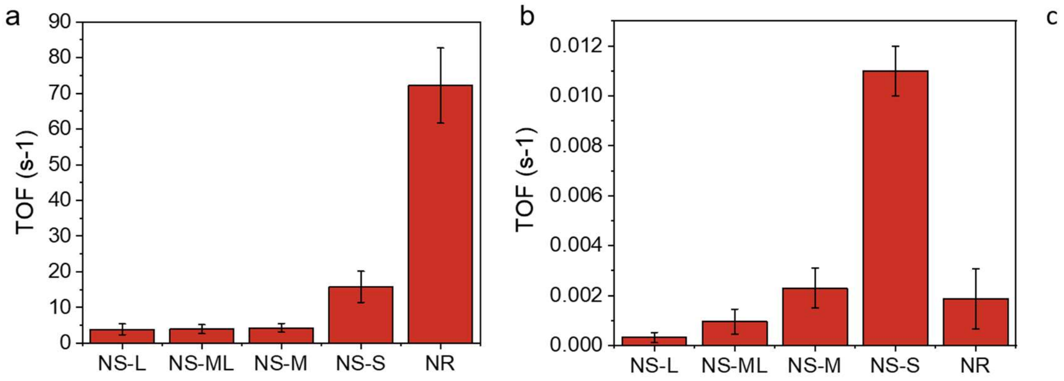

Supplementary Figure 17| TOFs of the CoOOH samples at the overpotential of 450 mV. TOFs were calculated by assuming (a) all the surface cobalt atoms and (b) all the bulk cobalt atoms were active. Standard errors for activities were calculated from the standard deviations from three measurements. Source data are provided as a Source Data file.

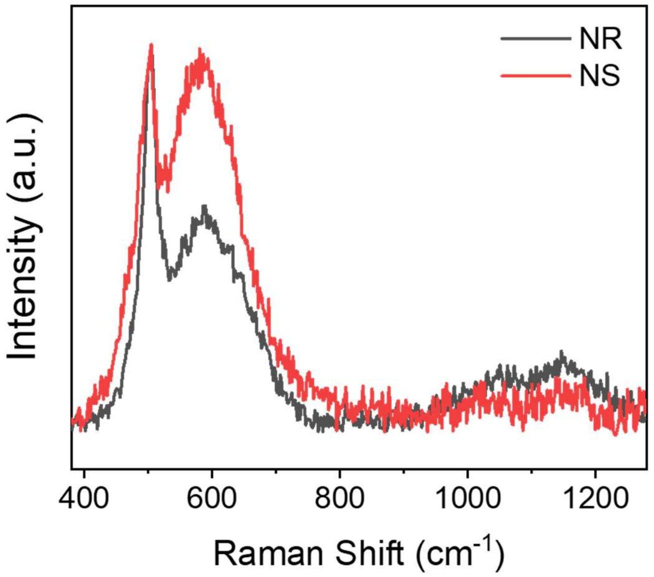

Supplementary Figure 18| Raman spectra of CoOOH NS (red) and NR (black). Both spectra were normalized to have the same E_g intensity of Co-O vibration. Source data are provided as a Source Data file.

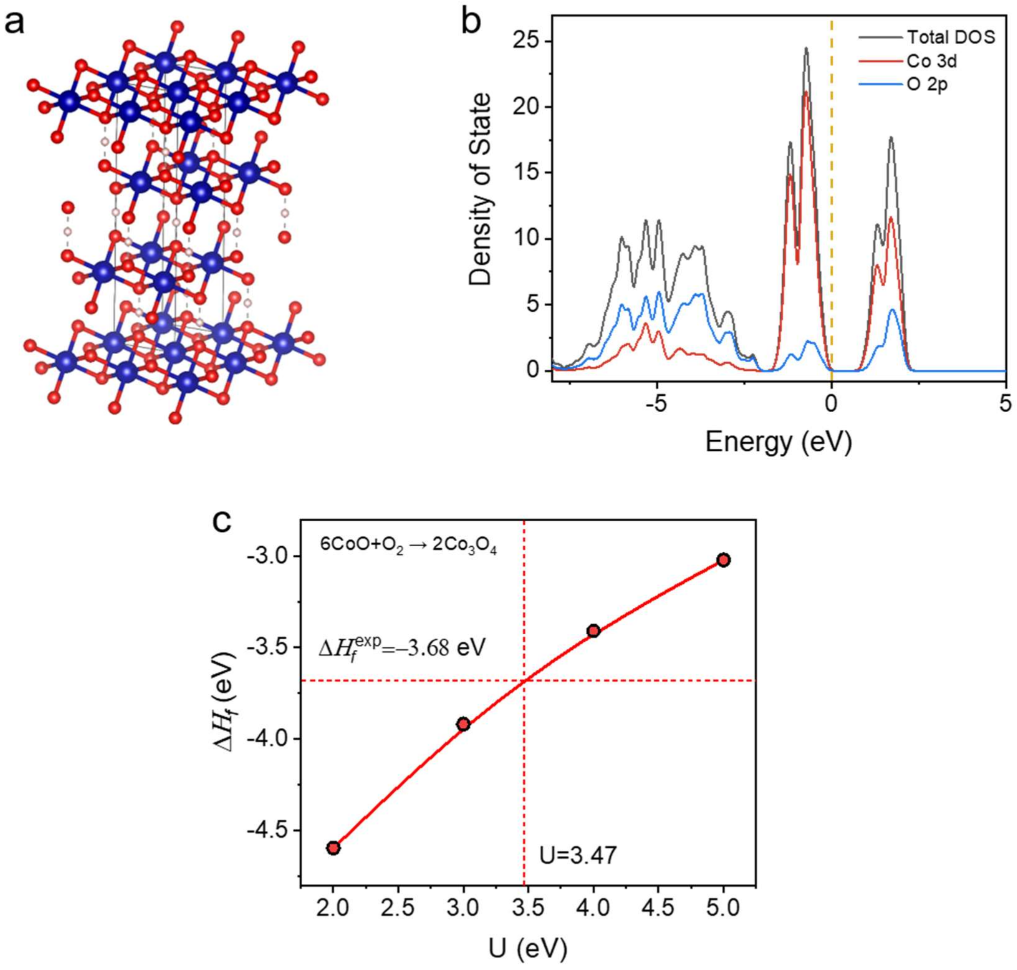

Supplementary Figure 19| Crystal model for theoretical calculation. (a) Optimized structure of $R\bar{3}m$ CoOOH crystal. (b) The density of states of β-CoOOH. (c) Enthalpy of oxidation (ΔH_f) as a function of U. Source data are provided as a Source Data file.

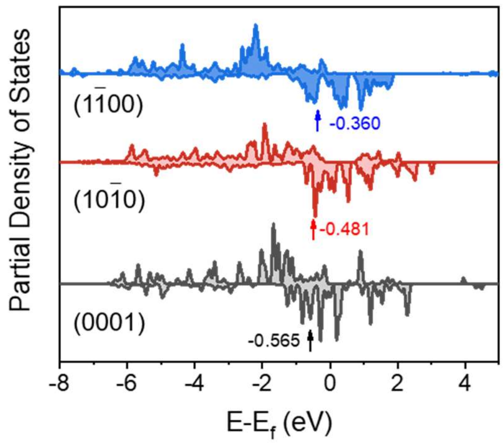

Supplementary Figure 20| The electron density of states for the Co$_{CUS}$ *3d* orbitals. Source data are provided as a Source Data file. (1$\bar{1}$00) is displayed in blue. (10$\bar{1}$0) is displayed in red and (0001) in black.

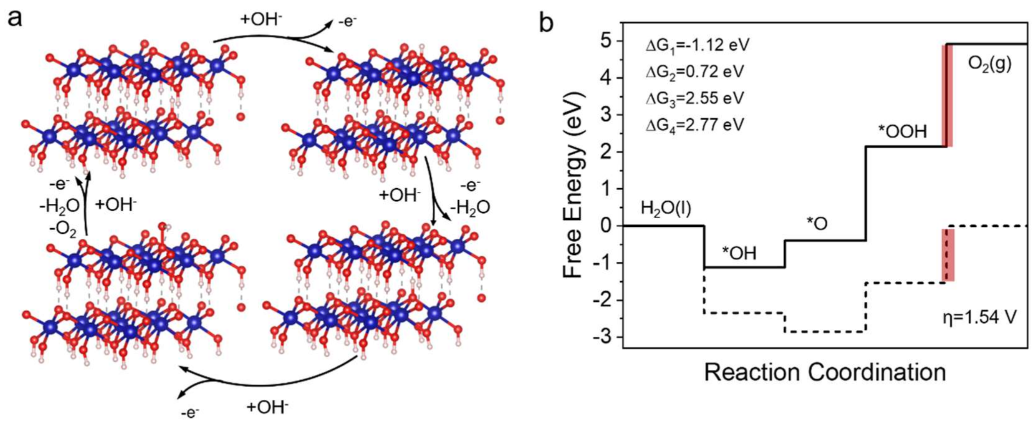

Supplementary Figure 21| Theoretical calculation of the OER on the (0001) surface with oxygen vacancy V_{O1} (PBE+U+D3). (a) Schematic diagram. (b) Free-energy landscape. Source data are provided as a Source Data file.

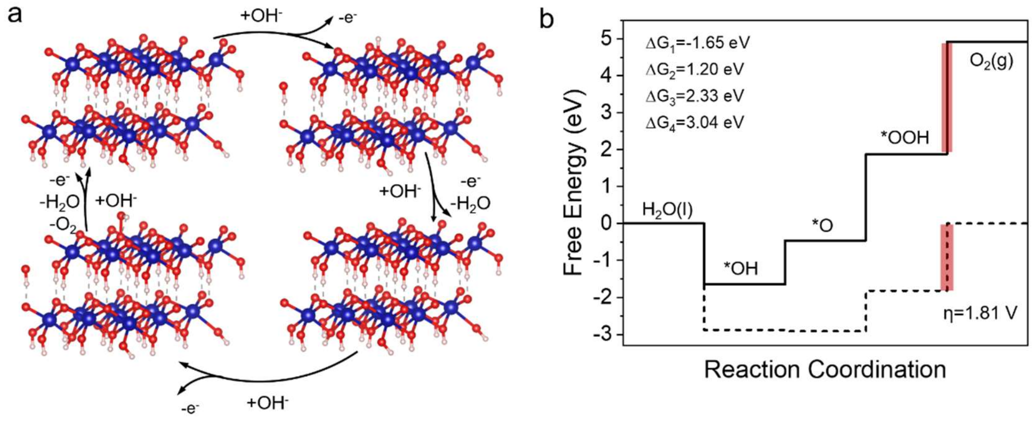

Supplementary Figure 22| Theoretical calculation of the OER on the (0001) surface with oxygen vacancy V_{O2} (PBE+U+D3). (a) Schematic diagram. (b) Free-energy landscape. Source data are provided as a Source Data file.

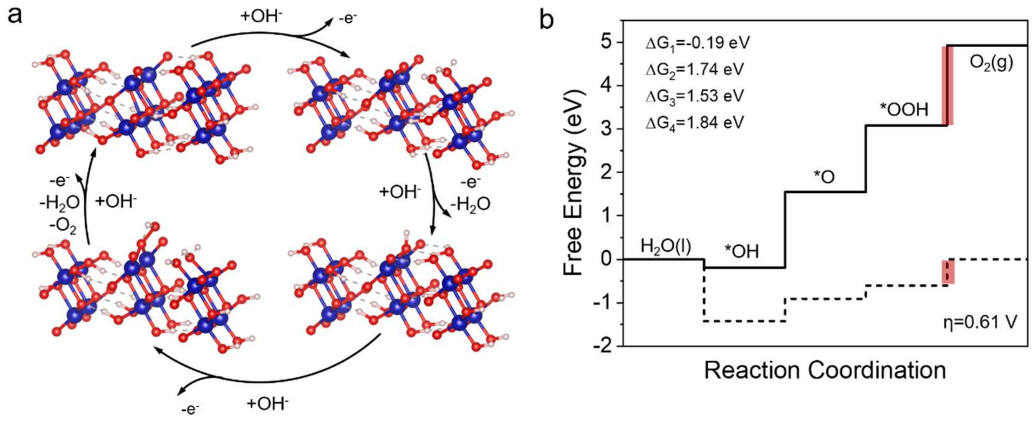

Supplementary Figure 23| Theoretical calculation of the OER on the $(10\bar{1}0)$ surface with oxygen vacancy V_{O1}. (a) Schematic diagram. (b) Free-energy landscape. Source data are provided as a Source Data file.

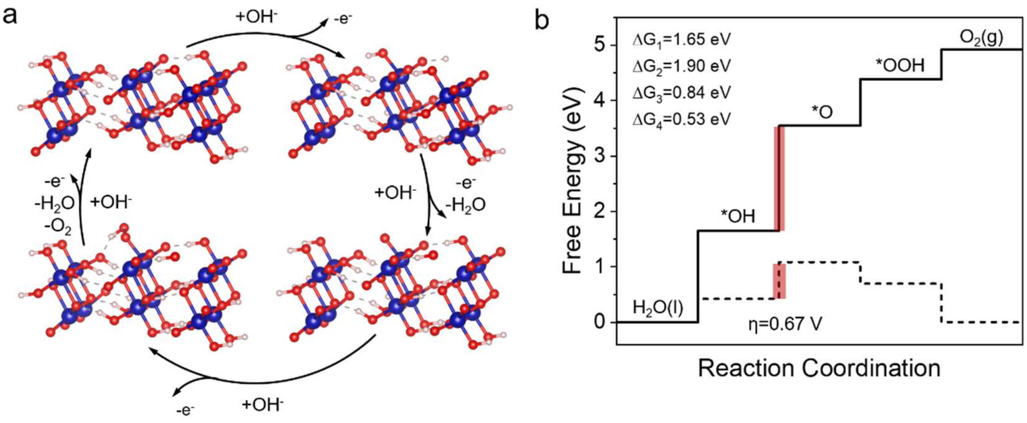

Supplementary Figure 24| Theoretical calculation of the OER on the (10$\bar{1}$0) surface with oxygen vacancy V_{O2}. (a) Schematic diagram. (b) Free-energy landscape. Source data are provided as a Source Data file.

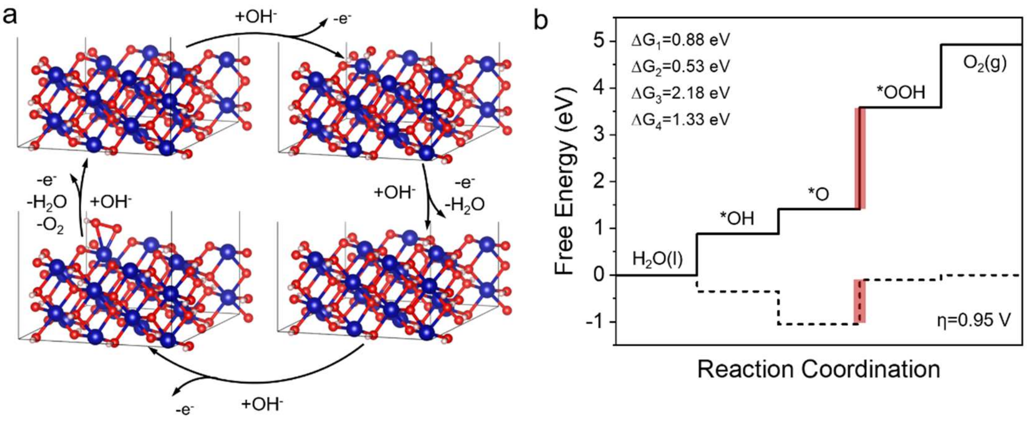

Supplementary Figure 25| Theoretical calculation of the OER on the $(1\bar{1}00)$ surface with oxygen vacancy V_{O1}. (a) Schematic diagram. (b) Free-energy landscape. Source data are provided as a Source Data file.

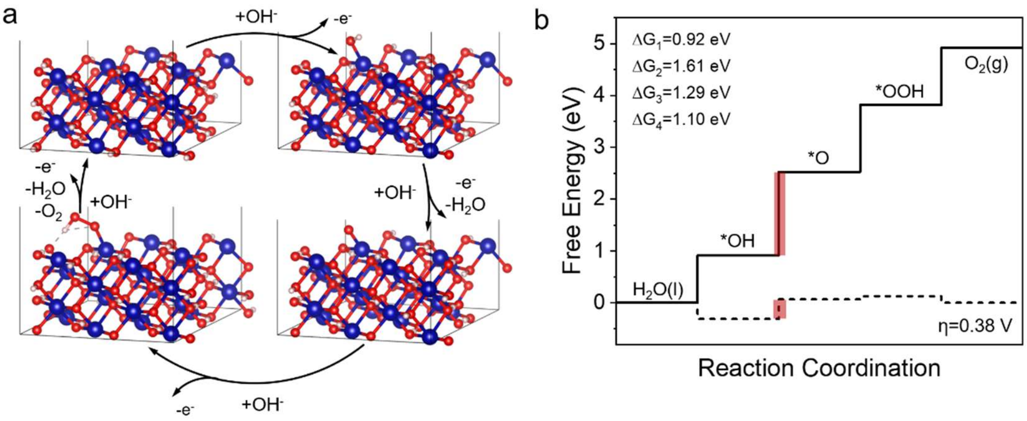

Supplementary Figure 26| Theoretical calculation of the OER on the (1$\bar{1}$00) surface with oxygen vacancy V_{O2}. (a) Schematic diagram. (b) Free-energy landscape. Source data are provided as a Source Data file.

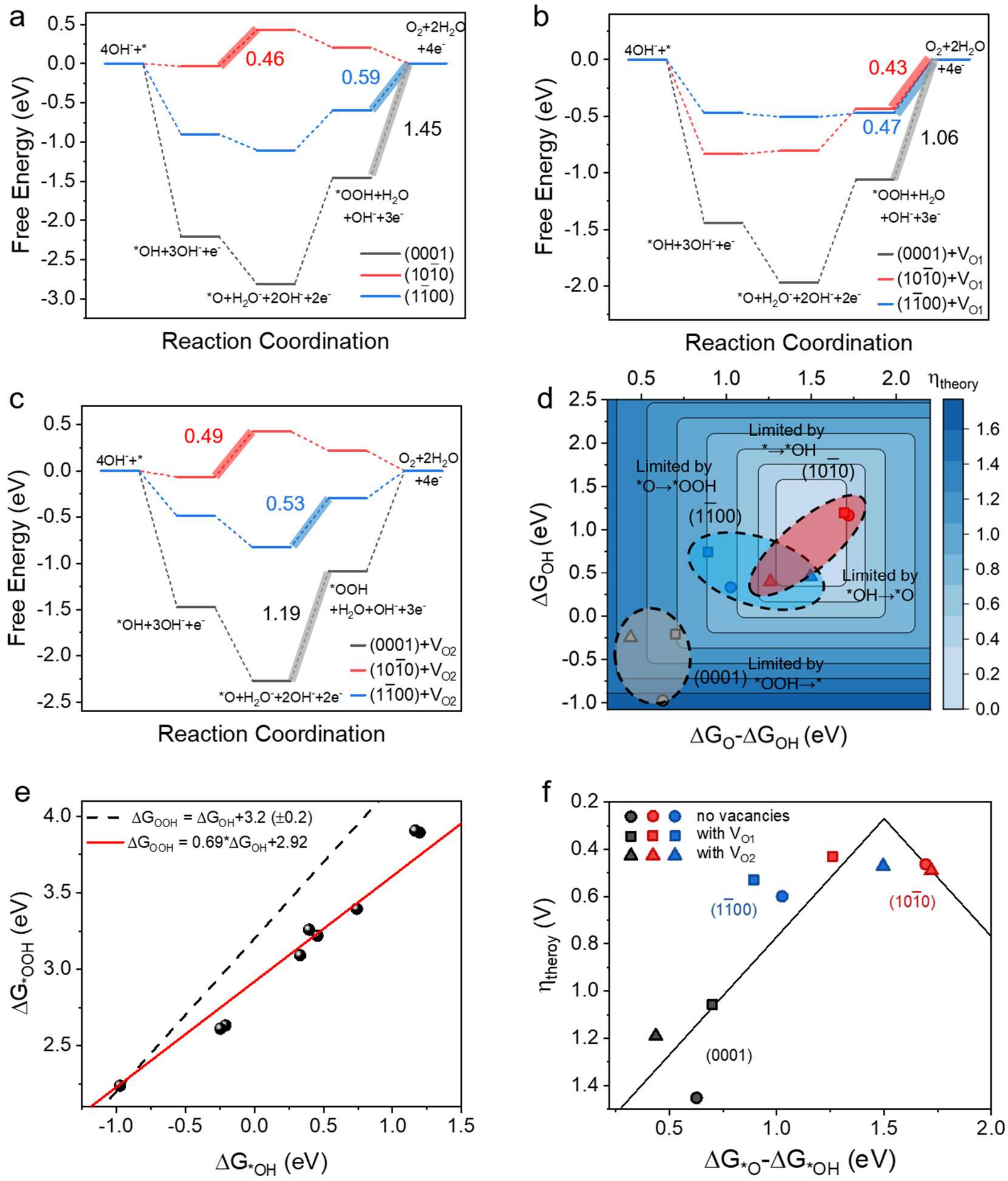

Supplementary Figure 27| Theoretical calculations on the PBE+D3 level. (a-c) Gibbs free energy diagrams on different facets of CoOOH (a) without oxygen vacancy, (b) with oxygen vacancy V_{O1}, and (c) with oxygen vacancy V_{O2}. (d) 2D Volcano plot. (e) Linear relationship between ΔE_{*OOH} and ΔE_{*OH}. (f) Volcano plot on the PBE+D3 level. Source data are provided as a Source Data file.

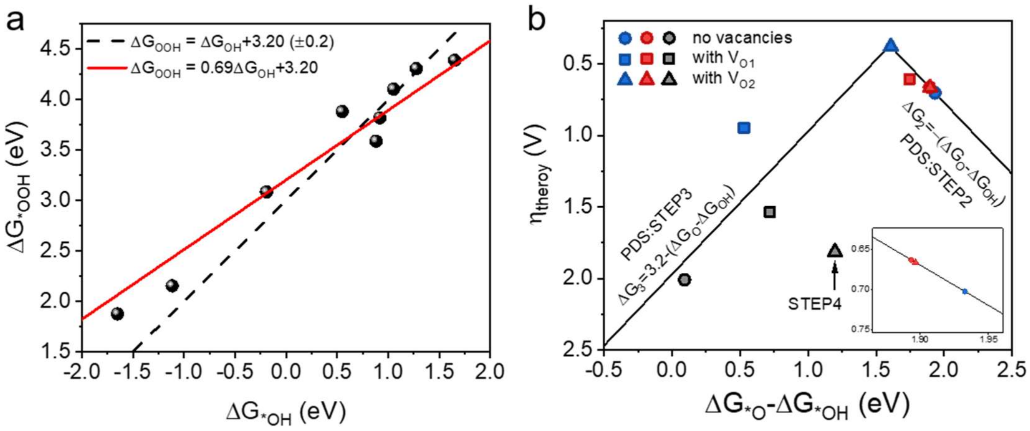

Supplementary Figure 28| Theoretical calculations on different facets of β-CoOOH. (a) Linear relationship between ΔE_{*OOH} and ΔE_{*OH} and (b) Volcano plot on the PBE+U+D3 level. Source data are provided as a Source Data file.

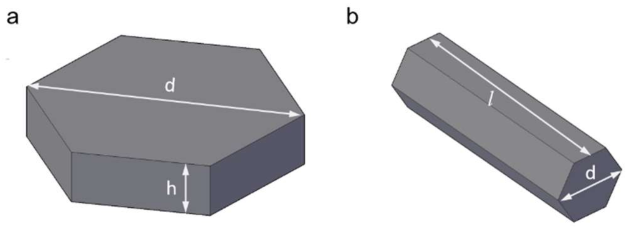

Supplementary Figure 29| Structural models for the calculation of surface area. (a) CoOOH NS and (b) CoOOH NR.

4 Supplementary Tables

Supplementary Table 1. The statistical data of particle sizes and roughness of CoOOH Samples. The particle sizes were based on measurements over an average of >100 particles.

**Sample Name**	**Average Basal Plane Size (μm)**	**Average Thickness/Length of Lateral Plane (μm)**	**Roughness (nm)**
CoOOH NS-L	3.76 ± 1.42	0.056 ± 0.08	0.62
CoOOH NS-ML	1.19 ± 0.37	0.062 ± 0.04	0.82
CoOOH NS-M	0.49 ± 0.08	0.063 ± 0.03	1.35
CoOOH NS-S	0.11 ± 0.04	0.058 ± 0.04	1.13
CoOOH NR	0.83 ± 0.24	5.825 ± 2.31	N.A.

Supplementary Table 2. The fitted parameters of Co K-edge EXAFS curves for CoOOH. (CN: coordination numbers; S_0^2: amplitude attenuation factors; R_{eff}: bond distance; σ^2: Debye-Waller factors)

**Sample**	**Bond**	**CN**	**S_0^2**	**R_{eff} (Å)**	**σ^2 （$*10^3$, Å^2 ）**
CoOOH NR	Co-O	5.7	0.07	1.90	4.9
CoOOH NS	Co-O	5.3	0.07	1.92	4.8

Supplementary Table 3. Fitting parameters for EIS data of NR and NS-ML.

Sample	Potential	R_{sol}	R_T	RC_T	CPE(C_T)	nCPE(C_T)	Cμ
	(V) vs. RHE	(Ω)	(Ω)	(Ω)	$\times 10^5$ ($F^{-1} \cdot s^{1-n}$)	--	(μF)
NR	1.68	9.27	1.51	44.41	1.87	0.92	6.66
	1.655	9.21	1.48	81.39	1.82	0.99	7.56
	1.63	9.25	1.54	213.65	1.28	0.97	9.27
	1.605	9.25	1.5	491.4	1.64	0.99	10.14
	1.58	9.2	1.5	1125.4 2	1.43	0.9	11.57

Sample	Potential	R_{sol}	R_T	R_{CT}	CPE(C_T)	nCPE(C_T)	Cμ
	(V) vs. RHE	(Ω)	(Ω)	(Ω)	$\times 10^6$ ($F^{-1} \cdot s^{1-n}$)	--	(μF)
NS-ML	1.68	9.3	7.22	61.1	8.521	0.93	4.91
	1.655	9.7	7.05	116.9	10.5	0.92	5.89
	1.63	8.71	6.99	256.1	10.5	0.92	6.13
	1.605	8.85	6.95	672.6	13.45	0.91	8.59
	1.58	8.73	6.92	1747	14.45	0.91	10.13

Supplementary Table 4. Comparison of TOFs of cobalt (oxy)hydroxides materials.

Catalysts	Electrode	TOF at η=450 mV(s^{-1})	Reference
CoOOH NS-S	GC	0.0110	This work
CoOOH NS-M	GC	0.0023	This work
CoOOH NS-ML	GC	0.0010	This work
CoOOH NS-L	GC	0.0004	This work
CoOOH NR	GC	0.0019	This work
$Co(OH)_2$	GC	0.0203	Mefford[4]
$Co(OH)_2$	GC	0.0149	Mefford[5]

Supplementary Table 5. Calculated bond length and dissociation energy of the surface Co-O bond.

Facet	Surface O	Bond length (Å)	Dissociation Energy (eV)
(0001)	O_{3c}	1.875	0.472
$(10\bar{1}0)$	O_{1c}	1.624	2.798
	O_{2c}	1.777	2.525
$(1\bar{1}00)$	O_{1c}	1.630	3.714
	O_{2c}	1.774	2.651

Supplementary Table 6. Calculated (PBE+D3/PBE+U+D3) Gibbs free energies of the OER on different facets without solvation correction.

Facets	Functional	Reaction Step (Formula)	ΔG / eV	Overpotential / V
(0001)	PBE+D3	* + 2H2O	--	1.60
		*OH + H2O + 1/2H2	-2.44	
		*O + H2O + H2	-2.71	
		*OOH + 3/2H2	-1.60	
		* + O2 + 2H2	0.00	
	PBE+U+D3	* + 2H2O	--	1.74
		*OH + H2O + 1/2H2	-2.60	
		*O + H2O + H2	-1.00	
		*OOH + 3/2H2	-1.74	
		* + O2 + 2H2	0.00	
$(10\bar{1}0)$	PBE+D3	* + 2H2O	--	0.42
		*OH + H2O + 1/2H2	-0.07	
		*O + H2O + H2	0.34	
		*OOH + 3/2H2	0.05	
		* + O2 + 2H2	0.00	
	PBE+U+D3	* + 2H2O	--	0.66
		*OH + H2O + 1/2H2	0.01	
		*O + H2O + H2	0.67	
		*OOH + 3/2H2	0.46	
		* + O2 + 2H2	0.00	
$(1\bar{1}00)$	PBE+D3	* + 2H2O	--	0.48
		*OH + H2O + 1/2H2	-0.68	
		*O + H2O + H2	-0.78	
		*OOH + 3/2H2	-0.48	
		* + O2 + 2H2	0.00	
	PBE+U+D3	* + 2H2O	--	0.78
		*OH + H2O + 1/2H2	-0.04	
		*O + H2O + H2	0.74	
		*OOH + 3/2H2	0.43	
		* + O2 + 2H2	0.00	

Supplementary Table 7. Bader charges q (in units of electrons) and local atomic magnetic moments m (in Units of μB) for the adsorbate sites of different facets.

Facet	slab (vac)		*OH		*O		*OOH	
	q	|m|	q	|m|	q	|m|	q	|m|
(0001)	1.38 (+2)	1.997	1.49 (+3)	0.001	1.48 (+4)	0.028	1.52 (+3)	0.003
$(10\bar{1}0)$	1.56 (+2)	2.137	1.61 (+4)	0.930	1.63 (+4)	1.081	1.58 (+3)	1.132
$(1\bar{1}00)$	1.47 (+2)	2.596	1.54 (+3~4)	0.14	1.58 (+3~4)	0.24	1.48 (+3~4)	1.490

Note： According to ligand-field theory, a Co^{2+} site shows either a high spin configuration with the spin multiplicity (S) of 3/2, or a low-spin state (S = 1/2), whereas a Co^{3+} site stays in a singlet spin state (S = 0) and a Co^{4+} is a low-spin state (S = 1/2).

Supplementary Table 8. The synthetic conditions and the sizes of the resultant $Co(OH)_2$ nanosheets. The particle sizes were based on measurements over an average of around 100 particles.

Sample Name	Alkaline Agents	Concentration of Alkaline Agents (mmol/L)	Concentration of Metal Salt (mmol/L)	Temperature of Reaction (°C)	Reaciton Time (h)	Average Particle Size (μm)
L	HMT	40	5	95	3	3.7
ML	HMT	90	5	95	3	1.2
M	NaOH	34	20	70	1	0.5
S	NaOH	60	20	70	1	0.1

References

1. Singh, C., Liberman, I., Shimoni, R., Ifraemov, R., Hod, I. Pristine versus pyrolyzed metal-organic framework-based oxygen evolution electrocatalysts: evaluation of intrinsic activity using electrochemical impedance spectroscopy. *J. Phys. Chem. Lett.* **10**, 3630-3636 (2019).

2. Xu, Z.P., Stevenson, G., Lu, C.Q., Lu, G.Q. Dispersion and size control of layered double hydroxide nanoparticles in aqueous solutions. *J. Mater. Chem. B* **110**, 16923-16929 (2006)

3. Yagi, S*., et al.* Covalency-reinforced oxygen evolution reaction catalyst. *Nat. Commun.* **6**, 8249 (2015).

4. Mefford, J. T. et al. Correlative operando microscopy of oxygen evolution electrocatalysts. *Nature* **593**, 67–73 (2021).

5. Mefford, J. T., Akbashev, A. R., Zhang, L. & Chueh, W. C. Electrochemical reactivity of faceted β-$Co(OH)_2$ single crystal platelet particles in alkaline electrolytes. *J. Phys. Chem. C* **123**, 18783–18794 (2019).
